# PCR cloning Intermediated Gibson assembly (PIG) for Constructing DNA Repair Templates in CRISPR-Cas9 Based Gene Editing

**DOI:** 10.17912/micropub.biology.000916

**Published:** 2023-08-18

**Authors:** Dongsheng Han, Scott Churcher, Jared T. Nordman

**Affiliations:** 1 Department of Biological Sciences, Vanderbilt University, Nashville, Tennessee, United States

## Abstract

The CRISPR-Cas9 gene-editing system has revolutionized genome engineering, allowing precise modifications to be made in a wide range of organisms. One significant challenge associated with CRISPR-Cas9 mediated gene editing is the construction of DNA repair templates containing homology arms, a screenable marker and a tag sequence of interest. Here, we present an efficient, two-step strategy to generate DNA repair templates in approximately one week, facilitating rapid and precise genome engineering applications.

**Figure 1.**
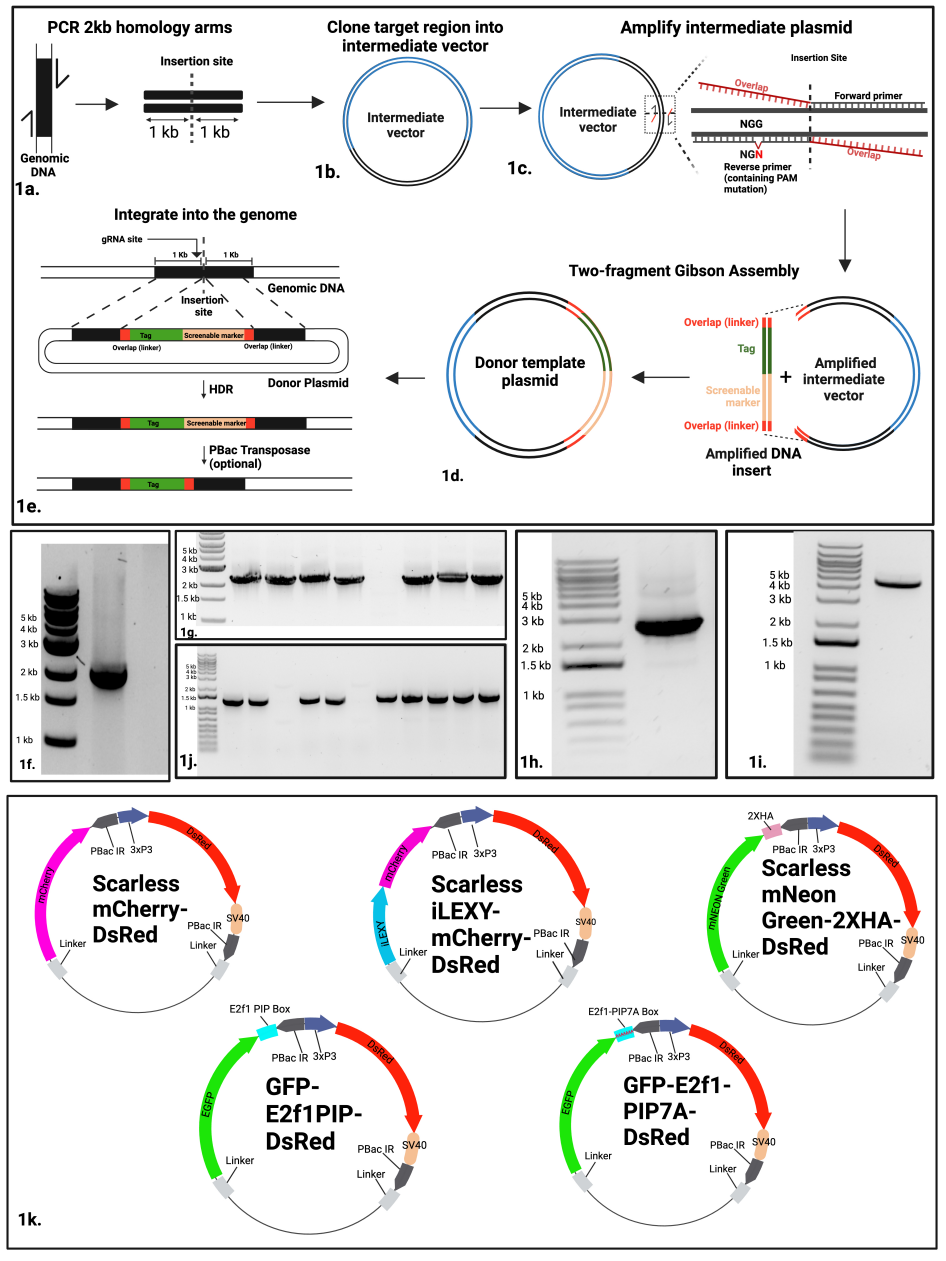
**Figure 1:** (a) PCR amplification of homology arms surrounding the insertion site of a gene of interest from genomic DNA, followed by (b) PCR cloning to generate an intermediate vector. (c) Primer design for the amplification of the intermediate vector for Gibson assembly, including the introduction of a PAM sequence mutation. (d) The amplified intermediate vector and insertion fragment come together in a single two-part Gibson assembly reaction to create the final donor template plasmid. To generate the insert fragment containing a tag sequence, screenable marker and overlap, one can either amplify the fragment from a pre-existing plasmid or have the fragment synthesized. (e) The HDR process integrating the repair template into the genome. The selectable marker can be removed by crossing with a PBac transposase-expressing fly line. To showcase this method, a 2 kb region of *CG42232* gene was amplified from genomic DNA (f). The 2 kb PCR product was cloned into the pMiniT 2.0 Linear plasmid using the NEB®PCR Cloning Kit and colony PCR was performed using the supplied primers (g). (h) The 2.5 kb mCherry-DsRed fragment was PCR amplified from the scarless mCherry-DsRed plasmid using primers creating the overlap for Gibson Assembly. (i) The 4.5 kb intermediate vector was PCR amplified from the insertion site using primers that create overlap with the insertion. (j) Colony PCR for Gibson reaction transformants from the final assembly using a pair of primers containing homology to the insert and intermediate vector, respectively. Positive transformants should give rise to a 1.3 kb PCR product. (k) Diagrams showing the available vectors containing various tags to assist the fly research community. All plasmids contain linker sequences for easy Gibson assembly, DsRed for easy selection and a tag sequence (mCherry, iLEXY-mCherry, mNeonGreen + 2xHA, EGFP-E2f PIP box, E2f-PIP7A/D62A).

## Description

The CRISPR-Cas9 gene-editing system has emerged as a powerful tool for precise genome editing in many organisms (Jinek et al., 2012; Ran et al., 2013) . The system relies on the formation of double-strand breaks (DSBs) in the target DNA, which are repaired by the cellular machinery through one of two major pathways: non-homologous end joining (NHEJ) or homology-directed repair (HDR) (Scully et al., 2019) . For precise gene-editing events, such as the introduction of specific mutations, insertions, or deletions, HDR is the preferred pathway (Sander & Joung, 2014) . HDR can be effectively used to introduce tag sequences into specific genetic regions of interest through the construction of DNA repair templates containing homology arms, an optional screenable marker and the chosen tag sequence (Gratz et al., 2014) . Cloning of recovery vectors, however, can be challenging and time-consuming, particularly when dealing with large genomic regions.

We have developed a streamlined approach for creating DNA repair templates for CRISPR-Cas9-based gene-editing systems. This method involves single-step PCR amplification of homology arms and subsequent cloning into an intermediate vector using a PCR cloning kit ( Figure 1a ). Next, the tag sequence and screenable marker (if desired) are PCR amplified creating a fragment with complementary overhangs to the homology arms ( Figure 1b ). Finally, Gibson Assembly is performed with the PCR-amplified intermediate vector and the DNA fragment containing a tag sequence, screenable marker, and overlap sequences (GGS linker) on both the 5’-terminus and 3’-terminus ( Figure 1d ) (Gibson et al., 2009) . Conveniently, the PCR primers used to amplify the intermediate vector for Gibson assembly mutate the PAM site to prevent CRISPR-Cas9 cleavage of the repaired region ( Figure 1c ).

Our method for generating DNA repair templates offers two major advantages over traditional techniques. First, it demonstrates a high success rate attributed to the efficiency of each step and the accessibility of commercial kits, ensuring a dependable two-piece Gibson process. The streamlined workflow only requires PCR amplification and a single two-piece Gibson Assembly reaction, thus minimizing the required cloning steps. Second, the versatility of this approach enables straightforward customization to accommodate diverse genomic regions, tag sequences, and optional screenable markers.

In this study, we outline our approach for constructing CRISPR-Cas9 DNA repair templates. As a proof of principle, we used this approach to generate a vector to insert the DsRed screenable marker and the red fluorescent protein mCherry within the *Drosophila melanogaster CG42232* gene (Gratz et al., 2013; Gratz, Harrison, et al., 2015; Gratz, Rubinstein, et al., 2015) . Initially, we PCR-amplified the genomic region (~2Kbp) surrounding the stop codon of the *CG42232* gene ( Figure 1f ) and cloned the PCR fragment into a vector using a PCR Cloning Kit (NEB). With ~1kb homology arms, the CRISPR-Cas9 system is reported to achieve an average insertion efficiency of 26% at multiple loci in Drosophila (Gratz, Rubinstein, et al., 2015) . We screened the clones and selected the correct clone based on size and verified it by sequencing, thus generating the intermediate vector ( Figure 1g ). Next, we PCR-amplified the DsRed-mCherry cassette from the mCherry-ScarlessDsRed plasmid ( Figure 1h ). During the PCR amplification of the intermediate vector, we designed primers that contained a mutated PAM site to simultaneously mutate the PAM sequence in the recovery vector to prevent cleavage of the edited genome ( Figure 1i ). All PCR products were examined using DNA gel electrophoresis and the products were gel purified. Lastly, we performed a two-piece Gibson Assembly with the PCR amplified fragment. To identify fully assembled plasmids, colonies were screened by colony PCR using primers that hybridized to the junction of the intermediate vector and amplified the insertion region ( Figure 1j ). Alternatively, primer pairs containing one primer on the insertion sequence and another primer that anneals with the vector can be used. Correct clones were sent for whole-vector sequencing (Plasmidsaurus).

We have expanded the tagging options for the fly research community by constructing five additional template vectors containing common tag sequences ( Figure 1k ). These vectors include mCherry, a red fluorescent protein for protein visualization; iLEXY-RFP, an optogenetic loss-of-function system that triggers rapid, efficient, and reversible protein translocation from the nucleus to the cytoplasm (Kögler et al., 2021); mNeonGreen + 2xHA, a combination of green fluorescence and affinity purification; EGFP-E2f PIP box, a fusion of PIP degron that directs protein degradation specifically in S phase of the cell cycle (Havens & Walter, 2009; Lee et al., 2010) and E2f-PIP7A/D62A, a mutated PIP degron that serves as a negative control for PIP degron studies. These vectors provide versatile tools to address diverse experimental questions and will be available by contacting the corresponding author. We note that additional tags can easily be swapped into any one of these vectors with a simple two-step Gibson assembly reaction. Similarly, the method can be easily adapted for N-terminal tagging by constructing homology arms around the start codon of the gene. Note that for N-terminal tags, following DsRed screening of engineered lines, an additional cross with a fly expressing PBac transposase is required to remove the DsRed cassette ( Figure 1e ). Alternatively, a tag can be inserted without a screenable marker and potential CRISPR lines can be screened by PCR and sequencing.

In summary, we present an efficient strategy for constructing DNA repair templates for CRISPR-Cas9-based gene-editing systems. This method overcomes current challenges associated with the inefficiency of generating repair templates containing homology arms, a selectable marker, and a tag sequence, thus offering a streamlined approach for rapid and precise genome engineering applications. Our method can be used to generate repair templates for any organism and nearly any tag or screenable marker can be swapped into repair vectors that already exist using a two-piece Gibson Assembly reaction.

## Methods

**PIG cloning Protocol:**

**Step 1: Generate the intermediate plasmid containing the target sequence**

1. Design primers using tools like NCBI Primer-BLAST to PCR clone the 2 kb genomic DNA region flanking the insertion site (1 kb upstream and 1kb downstream).

2. Amplify the ~2 kb genomic DNA sequence using a high-fidelity PCR polymerase master mix (e.g., NEB Q5). Verify the PCR products by agarose gel electrophoresis.

3. Purify the PCR product using a PCR clean-up kit or perform gel extraction if non-specific bands are present (e.g., TOYOBO MagExtractor).

4. Clone the ~2 kb genomic DNA sequence into the intermediate vector using a suitable cloning kit (e.g., NEB PCR Cloning Kit) following the manufacturer's protocol.

Screen the clones using colony PCR. Validate the insert by restriction digestion and verify the entire plasmid sequence at Plasmidsaurus or another suitable method.

**Step 2: Insert tag and screenable marker into the intermediate vector**

1. Design primers for Gibson assembly using online tools like NEBuilder. Ensure that the primer sequences of the intermediate vector start immediately following the insertion site (forward primer) and end just prior to the insertion site (reverse primer) ( Figure 1 ). If the gRNA PAM sequence is within 35 bp of the target site, consider incorporating a PAM site mutation (NGG to NGN) during primer design.

2. Amplify the two fragments (the tag with screenable marker and the intermediate vector) using a high-fidelity PCR polymerase master mix (e.g., NEB Q5). Analyze the PCR products on an agarose gel. Purify the PCR products using a PCR clean-up kit or perform gel extraction if non-specific bands are present. Measure the DNA concentration using a nanodrop or Qbit.

3. Perform two-fragment Gibson Assembly using NEB Gibson Assembly Master Mix as per the manufacturer's instructions. Briefly, combine 100ng of the intermediate vector PCR product with a 2-fold molar excess of the insert, calculated using the online tool NEBioCalculator. For inserts smaller than 200 bp, use a 5-fold molar excess. Incubate the reaction at 50°C for 60 minutes. Transform the resulting assembly product into competent DH5α *E. coli* cells and plate them on selective agar plates.

4. Screen transformants to verify the correct assembly by conducting colony PCR and diagnostic restriction enzyme digestion candidate transformants. Confirm the desired construct by whole-plasmid sequencing of selected transformants to ensure accurate vector construction.

## Reagents

**Table:** 

CG42232 Stop codon 2 kb Forward primer	AAGCCTAAGAGTCCTTCAGG
CG42232 Stop codon 2 kb Reverse primer	GGACATATCGACTTGCAGGA
Intermediate vector Gibson Forward Primer	ggtggttcaggaggttccaaCCCATTTCGGTGGGATACCGTTGAAT
Intermediate vector Gibson Reverse Primer	ccgcggccgctaaattcaattcgCTTTCGTCGTGATCGAGTTGCGCGCTGTGGTGC
mCherry_DsRed_Gibson_Forward primer	gcaccacagcgcgcaactcgatcacgacgaaaCGAATTGAATTTAGCGGCCG
mCherry_DsRed_ Gibson_Reverse Primer	attcaacggtatcccaccgaaatgggTTGGAACCTCCTGAACCACC
Colony screen forward primer	GAGGATCTGGACGACGAAAT
Colony screen reverse primer	TGTTGACGTTGTAGGCGCCG
NEB PCR cloning kit colony screen forward primer	ACCTGCCAACCAAAGCGAGAAC
NEB PCR cloning kit colony screen reverse primer	TCAGGGTTATTGTCTCATGAGCG
pScarlessHD-sfGFP-DsRed	Addgene #80811
NEB Q5® High-Fidelity 2X Master Mix	M0494
NEB® PCR Cloning Kit	E1202S
NEB Gibson Assembly® Master Mix	E2611S
SYBR™ Safe DNA gel stain	S33102
NEB® 1 kb DNA ladder	N3232S
TOYOBO MagExtractor	NPK-601
